# Retention and deformation of the blue phases in liquid crystalline elastomers

**DOI:** 10.1038/s41467-021-25112-6

**Published:** 2021-08-13

**Authors:** Kyle R. Schlafmann, Timothy J. White

**Affiliations:** 1 Department of Chemical and Biological Engineering, University of Colorado, Boulder, CO USA; 2 Department of Materials Science and Engineering, University of Colorado, Boulder, CO USA

**Keywords:** Liquid crystals, Liquid crystals

## Abstract

The blue phases are observed in highly chiral liquid crystalline compositions that nascently organize into a three-dimensional, crystalline nanostructure. The periodicity of the unit cell lattice spacing is on the order of the wavelength of visible light and accordingly, the blue phases exhibit a selective reflection as a photonic crystal. Here, we detail the synthesis of liquid crystalline elastomers that retain blue phase I, blue phase II, and blue phase III. The mechanical properties and optical reconfiguration via deformation of retained blue phases are contrasted to the cholesteric phase in fully solid elastomers with glass transition temperatures below room temperature. Mechanical deformation and chemical swelling of the lightly crosslinked polymer networks induces lattice asymmetry in the blue phase evident in the tuning of the selective reflection. The lattice periodicity of the blue phase elastomer is minimally affected by temperature. The oblique lattice planes of the blue phase tilt and red-shift in response to mechanical deformation. The retention of the blue phases in fully solid, elastomeric films could enable functional implementations in photonics, sensing, and energy applications.

## Abstract (web summary)

Permanently retaining liquid crystalline blue phases in a solid matrix warrants many prospective applications in photonics. Schlafmann and White are able to make a blue phase rubber that retains a crystalline structure upon deformation and responds with angle-dependent color changes.

## Introduction

Liquid crystalline elastomers (LCEs) are compelling responsive materials^1^. Recently, they have drawn considerable research interest for their utility as machine-like mechanical actuators^2^ as well as the spatial programmability of their material properties^3^. Analogous to small-molecule liquid crystalline systems, which have been prevalent in display applications for some time, LCEs also are relevant to a variety of optical and photonic applications including solid-state optical sensing^4^, tunable diffraction^5^, and adaptive lasing^6^.

Here, we detail an approach to prepare fully solid photonic materials that are robust, yet elastic and capable of dynamic reconfiguration. The fluidic nature of small-molecule liquid crystalline systems enables the response time and performance of liquid crystal displays^7^. In certain functional implementations, the realization of dynamic optical response in fully solid films is particularly desirable. Further, as evident in biological systems, dynamic reconfiguration of solid skins naturally induces multifunctionality even under ambient conditions^8^. Ranging from regulated structural color and diffuse reflectance in cephalopods^9, 10^ to glare-reducing corneal nipple arrays on nocturnal insect eyes^11, 12^ and photonic crystalline structures responsible for coloring in butterfly wing scales^13, 14^, the natural environment provides countless examples of dynamic light control. Many synthetic material systems have incorporated bio-inspired mechanisms to imitate their photonic properties for technological applications^15^.

Multidimensional photonic crystals such as those observed in the structural coloration in certain animals are of particular interest for optical applications such as nonlinear optics and waveguides^16^. These unique periodic nanostructures exhibit photonic band gaps in multiple propagation directions. Three-dimensional photonic crystals are nanoscale emulators of crystal structures typically observed in atomic lattices. Nanostructures such as those arising from inverse opals or lithographic deposition exhibit three-dimensional photonic crystallinity, however, the intensive fabrication of such materials is complex and costly^17^. The optics and photonics community has increasingly looked towards self-assembly and liquid crystals as light-manipulating media. Liquid crystalline materials can be formulated to adopt a catalog of anisotropic phases. Chiral liquid crystalline phases in particular exhibit photonic band gaps due to their periodic anisotropy^18^.

The liquid crystalline blue phases are a subset of liquid crystalline phases in which calamitic mesogens align in a double-twist morphology. The blue phases are frustrated phases and the nanostructure of these materials are a combination of defect packing and double-twist molecular arrangement of cylinders on the order of 100 nm in diameter^19^. These cylinders stack upon one another to generate cubic lattices stabilized by periodic disclinations. Blue phase I (BPI) organizes as a body-centered cubic (bcc) lattice, blue phase II (BPII) achieves a simple cubic (sc) lattice, and blue phase III (also called the “blue fog”) is largely amorphous. In this way, BPI and BPII are three-dimensional photonic crystals that are selectively reflective to visible light^19^.

The cubic blue phases have been extensively studied in non-cross-linked low molar mass systems^20–27^. The primary motivation for these studies is the potential for fast electro-optical switching properties in devices that do not need alignment layers or precise thickness control^28^. They have also been retained in free-standing glassy polymers upon cross-linking within the thermotropic phase window^29–34^. These polymers exhibit largely static photonic properties due to their high cross-link density, however recently the selective reflections of these materials have been sensitized to humidity and pH through supramolecular bonds within the network^33^ and to small amounts of mechanical deformation^34^. Polymer-stabilized blue-phase gels (32 wt.% polymerizable media) exhibit electro-optical response, along with limited photonic sensitivity to mechanical deformation^35^. However, the liquid crystalline blue phases have not been retained in truly elastomeric polymer networks with a glass transition below room temperature (*T*_g_ <20 °C).

We have reported a straightforward and scalable approach to prepare well-aligned cholesteric liquid crystal elastomers (CLCEs) in the planar orientation composed of main-chain chiral mesogens^36^. Here, enabled by similar reaction chemistry, we prepare liquid crystalline elastomers that retain the blue phases upon photopolymerization. We characterize changes in the lattice spacing to mechanical deformation, heat, and chemical exposure.

## Results and discussion

### Retention of blue phases

Historically, liquid crystalline elastomers (LCEs) have been prepared by two-stage polymerization reactions in which alignment is enforced in the second stage by mechanical force. The preparation of hierarchical liquid crystalline phases, such as the cholesteric or blue phases, are not readily amenable to these processes. The helicoidal nanostructure of the cholesteric phase has been retained in LCEs by mechanical alignment coupled with anisotropic deswelling through either centrifugation^37–39^ and/or anisotropic deswelling^40^. However, the intricate, three-dimensional nanostructure of the blue phases has not been retained in LCEs by these methods. A recent report^36^ details the development of a materials chemistry to prepare LCEs that originate from the desired chiral phase that are conducive to surface-enforced alignment and prepared from a one-step reaction. This chemistry was utilized to prepare and retain the cholesteric phase in LCEs of high optical quality (low haze) and considerable thermochromism.

**Fig. 1:**
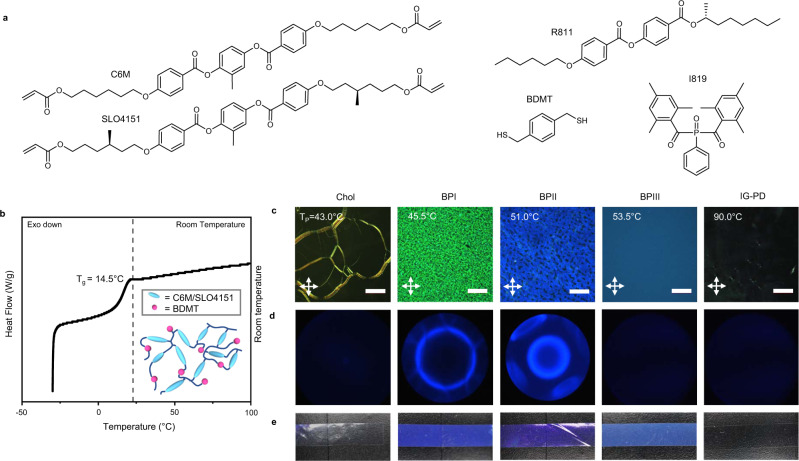
Blue-Phase LCE formulation and phase retention. **a** Chemical structures of monomers. **b** DSC thermogram illustrating glass transition of the polymer network, independent of the phase retained. Inset) the LCE forms a hyperbranched polymer network. **c** Polarized optical micrograph textures (in reflection mode) of the LCE. Scale bars are 100 μm. Images are captured at room temperature after each phase is retained via photopolymerized at the polymerization temperature (TP). From left to right: cholesteric phase (Chol), blue phase I (BPI), blue phase II (BPII), blue phase III (BPIII), and isotropic genesis polydomain nematic (IG-PD). **d** Kossel diagram (440 nm) of free-standing elastomers retaining the stated phase confirming cubic structures for blue phase I (body-centered cubic [110]) and blue phase II (simple cubic [100]). **e** Photographs of LCE retaining either Chol, BPI, BPII, BPIII, or IG-PD phase, unpolarized illumination.

The blue phases are observed in formulations that have a high concentration of chiral species. The liquid crystalline molecules organize in cubic, nanostructured phases. The periodicity of the blue phases is defined by crystalline packing within unit cells. The blue phases are typically observed in very narrow temperature ranges, which can complicate the retention of these phases in polymer networks. Here, we report the synthesis of LCEs that retain blue phase I (BPI), blue phase II (BPII), and blue phase III (BPIII). The composition is based on a mixture of the liquid crystalline diacrylate C6M and chiral liquid crystalline diacrylate SLO4151 (Fig. 1a). The nonreactive chiral dopant R811 is added at 15 wt%. The addition of R811 both broadens the temperature range of the blue phases before polymerization (Supplementary Fig. 1) and enables the formation of BPII and BPIII in these mixtures. Notably, blue-phase LCEs can be prepared without the presence of the nonreactive chiral dopant but over very narrow temperature windows. We employ a one-step free-radical photopolymerization reaction between the acrylate monomers and 14.2 wt% of the dithiol, BDMT. BDMT can react as both a chain extender and chain transfer agent^41^. Polymerization is photoinitiated with Omnirad 819. A representative DSC trace of the LCE is illustrated in Fig. 1b (individual traces found in Supplementary Fig. 2). All the LCE detailed in this examination have below-room temperature glass transition temperatures (*T*_g_) of ~15 °C. The acrylate-thiol chain transfer reactions suppress the cross-link density of the acrylate homopolymerization by producing a highly branched polymer network architecture illustrated in the inset to Fig. 1b^41^.

The formulation to prepare LCEs with 15 wt% R811, 14.2 wt% BDMT, liquid crystalline monomers, and photoinitiator exhibits five liquid crystalline phases before polymerization. Accordingly, this study is uniquely able to prepare LCEs in the isotropic genesis polydomain nematic, cholesteric, BPI, BPII, and BPIII phases simply by varying the polymerization temperature. Upon polymerization, the LCEs retain the characteristic birefringent textures of these phases when imaged with polarized optical microscopy (POM, Fig. 1c). To confirm the retention of cubic periodicity in LCEs, we undertook so-called Kossel diffractive measurements^24^. As expected, the cholesteric, BPIII, and polydomain nematic phases do not exhibit a diffraction pattern (Fig. 1d). However, the cubic orientation of BPI and BPII is evident (Fig. 1d). The photographs of the LCE retaining the cholesteric, BPI, and BPII illustrate the retention of the selective reflection associated with these phases. A hazy blue texture indicates the amorphous BPIII phase, which is not cubic (evident in Fig. 1d) and does not exhibit a coherent reflection. Extraction of the nonreactive chiral dopant from the LCE results in a slight blue shift in reflection color, evident in the images of the free-standing LCE films (Fig. 1e) when compared to the POM images in Fig. 1c (see also Supplementary Fig. 3). Gel fraction measurements confirm the complete incorporation of the reactive components and removal of the nonreactive chiral dopant.

### Optical reconfiguration

**Fig. 2:**
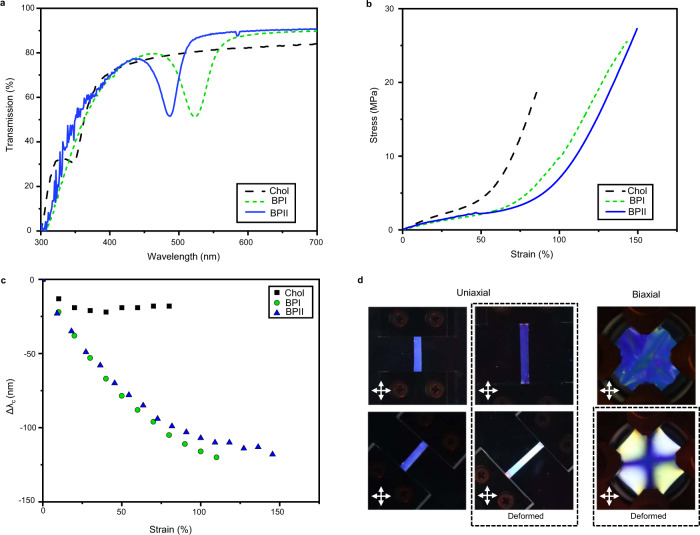
Optical properties and effects due to mechanical deformation. **a** Transmission spectra of elastomers retaining the cholesteric phase, blue phase I (BPI), and blue phase II (BPII) showing selective reflection bands as measured through incident light to the surface of the elastomer. **b** Stress–strain behavior of cholesteric, blue phase I, and blue-phase II elastomers. **c** Change in selective reflection of elastomers retaining the cholesteric phase, blue phase I (BPI) and blue phase II (BPII) as a function of uniaxial strain. **d** Birefringence patterns induced by uniaxial or biaxial mechanical deformation of blue-phase I elastomer. Observed as photographs through crossed polarizers.

Informed and enabled by the retention of the blue phases in fully solid and elastomeric LCE, we focus the remainder of this examination on LCE retaining the BPI and BPII phases. The selective reflection of unperturbed BPI and BPII LCE, visualized in the photographs in Fig. 1e, were quantified with UV/Vis spectroscopy (Fig. 2a). These spectra are contrasted to the LCE retaining the cholesteric phase, which due to the large concentration of chiral monomer and chiral dopant used to prepare these LCE, exhibits a selective reflection below 350 nm (appearing transparent in Fig. 1e). UV/Vis spectra for BPIII LCE and polydomain nematic are shown in Supplementary Fig. 4. The mechanical deformation of LCE retaining BPI, BPII, and the cholesteric phase are evident in Fig. 2b. The stress–strain responses of BPI LCE and BPII LCE are nonlinear between 25 and 70% strain before transitioning to a strain hardening region. Comparatively, the deformation of the cholesteric LCE (CLCE) retained from the identical composition exhibits strain hardening around 50% strain. The deformation of BPI, BPII, and BPIII LCE resembles the soft elasticity typically observed in isotropic genesis polydomain nematic LCE (Supplementary Fig. 5). We attribute this similarity to the double-twist cubic nanostructure which is macroscopically isotropic in three dimensions. Comparatively, while the director in CLCE introduces effective isotropic mechanical properties in the *x*–*y* plane, the planar organization of these materials does differentiate the mechanical properties in the *z* axis (through the thickness). Thus, we conclude the phase of the LCE is the primary differentiator in stress–strain behavior reported here.

The mechanooptical response of the deformation of BPI and BPII was examined by UV/Vis spectroscopy during an applied load. The selective reflection (${{{\lambda}}}_{h{kl}}$) of the cubic blue phases (BPI and BPII) is defined by:

1 $${{{\lambda }}}_{{hkl}}=\frac{2\bar{n}a}{\sqrt{{h}^{2}+{k}^{2}+{l}^{2}}}$$

where $\bar{n}$ is the average refractive index, $a$ is the lattice constant, and $h,k,l$ are the Miller indices. Uniaxial deformation lengthens the material in the loading axis while reducing the width of the film and more importantly, the material thickness. Again, the optical properties of BPI and BPII LCE are derived from periodic cubic nanostructures. The directional bias introduced by mechanical load affects the lattice spacing within the BPI and BPII which cause the LCE retaining these phases to undergo a sizable blue shift in the primary reflection. The magnitude of this shift (120 nm) is greater than that observed for the CLCE polymerized from the same formulation. The reflection of the BPI LCE is associated with the [110] plane of a body-centered cubic lattice while the reflection of the BPII LCE is associated with the [100] plane of a simple cubic lattice. The blue shift in the deformation of a BPI LCE and BPII LCE are visually evident in Fig. 2c. Note, due to the position of the [100] reflection of BPII LCE, deformation nearly immediately shifts the reflection into the ultraviolet (UV). Analogous to low molar mass blue phase materials, the BPI and BPII LCEs maintain a circularly polarized reflection that matches the handedness of the chiral dopant used. To deformation of 50% strain, the reflection of both BPI and BPII LCE becomes unpolarized (Supplementary Fig. 6). A conversion from polarized to unpolarized reflection has been predicted^42^ and observed^43^ in CLCE, owing to asymmetry in the period distribution of refractive index associated with thickness-dependent (orientation-dependent) reorientation to the strain axis. The biaxial strain of BPLCE results in an alternating pattern of yellow and blue regions, indicating a complex rearrangement of the mesogens coupled with the photonic properties of the blue phase. The physical reason for this distinctive texture is subject to further study.

**Fig. 3:**
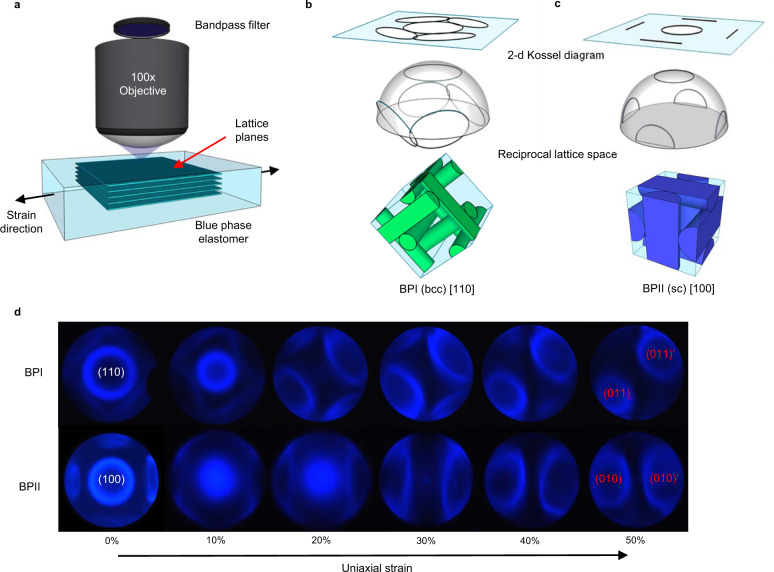
Blue-phase LCE lattice deformation (Kossel diagram analysis). **a** Kossel diagram capture apparatus. A polarized optical microscope was employed with a Bertrand lens, a ×100 objective lens, and 440 nm bandpass filter. **b** Top: Theorized Kossel diagram of [110] oriented blue phase I. Middle: Reciprocal lattice space of [110] oriented blue phase I. Bottom: Unit cell of [110] oriented blue phase I. **c** Top: Theorized Kossel diagram of [100] oriented blue phase II. Middle: Reciprocal lattice space of [100] oriented blue phase II. Bottom: Unit cell of [100] oriented blue phase II. **d** Kossel diagrams of blue phase I (BPI) and blue phase II (BPII) elastomers as a function of (horizontal) uniaxial strain, showing unit cell reconfiguration.

The influence of uniaxial deformation of BPI and BPII LCE to introducing cubic asymmetry is further elucidated by Kossel imaging. In this method, monochromatic light (440 nm) is incident upon the blue-phase LCE through a high numerical aperture objective (Fig. 3a). The rear focal plane is observed using a Bertrand lens. The incident light exhibits a characteristic diffraction pattern as it passes through the cubic lattice of the blue-phase LCE. The two-dimensional projection of the reciprocal lattice space is captured (Fig. 3b, c).

Evident in Fig. 3d, in the undeformed state, the BPI LCE (bcc) maintains [110] lattice alignment in the viewing direction with Kossel lines corresponding to the adjacent [011] and [101] planes at approximately the expected angle of 60° from the viewing plane. As the BPI LCE is subjected to uniaxial load, the circle corresponding to the [110] plane decreases in diameter. The decrease indicates that the lattice spacing of the [110] plane is decreasing, which affects the periodicity of the nanostructure. Reduction of the normal-incidence reflection wavelength to less than the wavelength of light used for the Kossel diagram prohibits diffraction. Thus, the central circle is lost around 20% strain. Simultaneously, the arc Kossel lines corresponding to the [011] planes become more circular with strain, while the Kossel lines corresponding to the [101] planes depart the field of view. We believe this confirms the [011] lattice planes are tilting toward the film surfaces during deformation. In addition, the periodicity of this plane does not decrease by the same magnitude as the vertically oriented [110] plane. This indicates that the uniaxial load induces lattice asymmetry.

The BPII LCE (sc) maintains [100] lattice alignment in the viewing direction with Kossel lines corresponding to the [010] and [001] planes observed at the expected angle of 90°. Similarly, the Kossel diagrams of BPII LCE during uniaxial deformation confirm that the lattice spacing becomes asymmetric to deformation. The simple cubic [100] periodicity decreases with strain. In an analogous fashion to the deformation-induced lattice tilting of BPII LCE, the [010] or [001] (whichever is oriented along the strain direction) tilts towards the film surface and maintains a periodicity much larger than that of the normal incidence [100] in the deformed state.

**Fig. 4:**
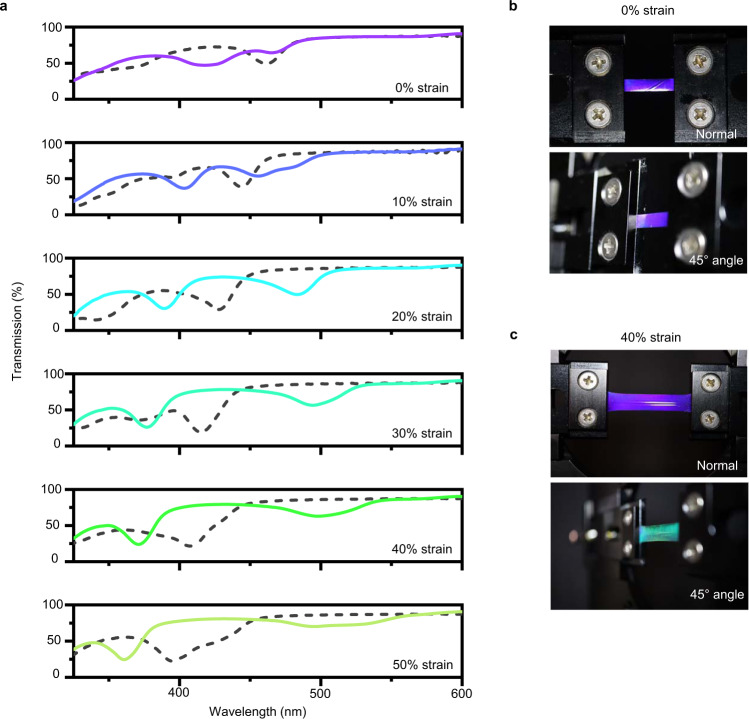
Optical confirmation of BPII LCE lattice asymmetry with deformation. **a** Transmission spectra of a BPII LCE with the surface of the film at normal incidence to the optical probe [dashed line] and the film angled 45° to the optical probe [solid lines] at uniaxial strains between 0% and 50%. **b** Photos of unstrained BPII LCE illuminated from camera direction. Top: Sample at normal incidence (bottom) 45° angle. **c** Photos of 40% strained BPII LCE illuminated from camera direction. Top: Sample at normal incidence (bottom) 45° angle.

Again, the liquid crystalline blue phases are cubic structures; their selective reflection originates from the periodicity of that cubic lattice. In that sense, each set of lattice planes act as a Bragg reflector which blue-shifts ∝ cos(*θ*) as the viewing angle increases. To further explore the spectral effect of mechanical deformation of the lattice, Fig. 4 measures the angular-dependent optical properties of a BPII LCE during deformation. Evident in Fig. 4a, at 0% strain the BPII LCE exhibits blue-shifting when rotated from orthogonal to the optical probe to angled 45°. At normal incidence (e.g., 0°), the reflection is primarily associated with the [100] plane. However, when probing the BPII LCE at 45° to the film surface, both the [100] and [010]/[001] (whichever is aligned along the long axis of the film) are observable. Both the [100] and [010]/[001] are each oriented at 90° angles to one another in a simple cubic lattice. Therefore, we would expect an undistorted BPII LCE to have a single, blue-shifted reflection band when measured at 45°, because the cos(*θ*) dependence is equivalent. Again, the extraction of the nonreactive chiral dopant after the preparation of these films may impart some residual stress that could be the source of the lattice tilt that may be the origin of the small reflection apparent at 460 nm. The photographs of the unstrained BPII LCE in Fig. 4b demonstrate a slight blue shift from blue to purple between the normal incidence and 45° sample angle.

As the sample is uniaxially deformed, the reflection associated with the [100] lattice plane at normal incidence begins to blue shift (as shown in Fig. 2b) due to the decreasing film thickness. The [100] reflection notch is blue-shifted further when the probe–sample–detector is at an acute angle, due to the Bragg condition. However, the [010]/[001] reflection at 45° BPII LCE begins to red-shift as the sample is strained. We attribute this to a combination of lattice tilt towards the surface of the film (as shown in Fig. 3d) and an increase in [010]/[001] periodicity. Our underlying assumption is that the total number of unit cells is unchanged in the cross-linked polymer network and thus, the redistribution of the volume of the LCE is accommodated by an asymmetry within the unit cells. The lattice constants of the unit cell become shorter in the [100] plane direction and longer in the strain-axis lattice plane direction. This effect is observable visually in the photographs in Fig. 4c. Of particular note, the red-shifted reflection of the [010]/[001] BPII LCE at 40% strain is apparent when imaged at an acute angle.

### Photopatternable lattice properties

**Fig. 5:**
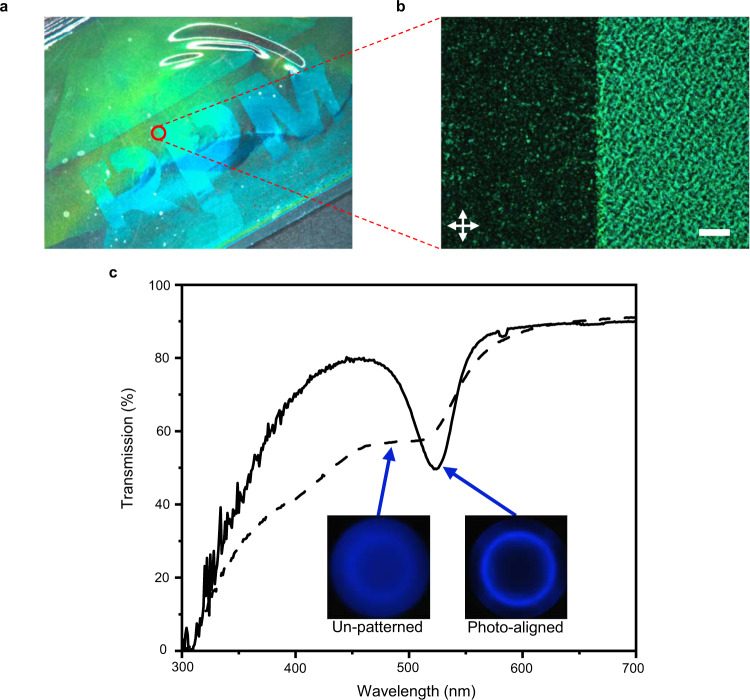
Patternable lattice alignment. **a** Demonstration of photopatterned blue-phase I lattice alignment. To prepare, a photomask was employed to selectively irradiate the photoalignment cell with 445 nm light. **b** Polarized optical micrograph texture imaged at the edge of the photopatterned region (scale bar 100 μm). **c** Transmission spectra of BPI LCE unpatterned and photoaligned regions. Kossel diagrams qualitatively assess lattice homogeneity.

The alignment of blue-phase materials has drawn considerable interest as of late due to the significant improvement in coherent reflective properties of the material. Without an alignment layer, BPI or BPII unit cells can orient randomly to the material surface and the phase is retained in a polycrystalline state. Due to natural differences in refractive indices, the polycrystalline blue phases can exhibit significant haze. Recent literature reports have documented the critical contribution of surface interaction to enabling alignment of large monocrystalline blue-phase domains^21^. Here, we demonstrate that the domain size and optical quality of the reflection of LCE retaining BPI or BPII can also be affected by variations in surface anchoring. Here, utilizing a commercially available photoalignment mixture PAAD-22 (BEAM Co.), an alignment cell was prepared and selectively irradiated with a 405 nm laser (Supplementary Fig. 7). As evident in Fig. 5a, the irradiated (patterned) region exhibits a bright green reflection while the masked (unpatterned) region appears hazier and exhibits a faint green reflection. The resolution and effectiveness of the photoalignment mixture is further explored with polarized optical microscopy. The stark difference in platelet size and degree of orientation is evident in Fig. 5b. While the aligned (irradiated) region is still polycrystalline (as evident by the dark regions in which the platelets reflect UV light), the degree of orientation is considerable. This discrepancy in reflection coherence is further exemplified in the transmission spectra and corresponding Kossel diffraction diagrams in Fig. 5c. While the photoaligned region of the BPI LCE exhibits a single, selective reflection at 520 nm, the unpatterned surface exhibits low transmission in the 350–500 nm range associated with random orientation of BPI unit cells. The hazy Kossel diffraction image is a further indication of the dispersity of crystalline orientation in the unpatterned regions.

### Stimuli response

**Fig. 6:**
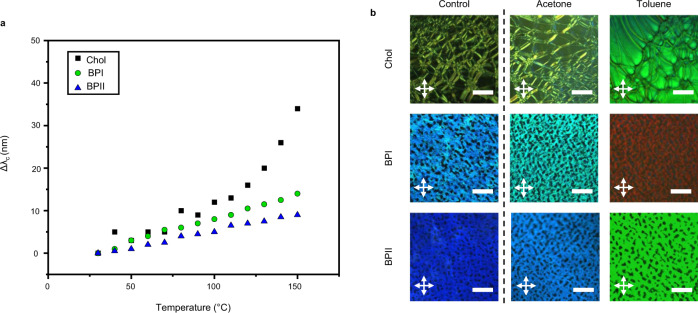
Optical response to chemical swelling and heat. **a** Selective reflection band wavelength of CLCE, BPI LCE, and BPII LCE as the samples are heated from room temperature to 150 °C. **b** Polarized optical micrograph textures observed in reflection mode of CLCE, BPI LCE, and BPII LCE (scale bar 100 μm) in response to swelling of organic solvents.

Upon heating, the BPI and BPII LCE exhibit a minimal change in reflection (Fig. 6a). This is distinctive from an LCE that retains the cholesteric phase, which exhibits upward of 200 nm shifts in selective reflection to changes in temperature^36^. As detailed above relating to the mechanical response, the effective macroscopic isotropy of BPI and BPII suppress the magnitude of the relative change in periodicity associated with the average thermal expansion coefficient of the polymer. Comparatively, LCEs retaining the CLC phase are isotropic in the *x*–*y* axes but anisotropic in the thickness, which results in a directional mechanical response to the thermotropic disruption of order.

Comparatively, swelling the BPI and BPII LCE with an organic solvent can yield sizable changes in coloration. The red-shift evident in the selective reflection of the POM micrographs in Fig. 6b are associated with a three-dimensional, symmetric increase in lattice spacing. A similar effect is observed in the solvent exposure of the initially UV-reflecting LCE retaining the CLC phase. The magnitude of the expansion in periodicity is solvent-dependent. The interaction between the solvent and the chemical environment within the elastomer matrix can be defined by solubility parameters, which quantify compatibility of dispersion forces, polarity, and hydrogen bonding^44^. Swelling with toluene (nonpolar) resulted in a comparatively larger uptake than the polar solvent acetone, which is qualitatively evident in the magnitude of the shift in reflection color. Exposure to dichloromethane resulted in even greater shifts into the near-infrared spectrum (see Supplementary Movie 1).

The branched, lightly cross-linked polymer network prepared from the liquid crystalline monomer mixture reported here can retain the three-dimensional, nanostructured architecture associated with either the body-centered cubic BPI, the simple cubic BPII, or the amorphous BPIII. Mechanical, thermal, and chemical stimuli uniquely affect the lattice constants and the associated optical properties of these free-standing and fully solid optical materials. Whether the stimuli exposure introduces symmetric (heat, swelling) deformation or asymmetric (mechanical force) deformation of the lattice of the blue phases, in all cases the distortion is reversible. The distinctive hierarchical nanostructure retained in the blue-phase LCE are unexplored but compelling for functional use as a lightweight, fully solid, and deformable optical materials of relevance in nonlinear optics, lasing, spectral imaging, and sensing.

## Methods

### Materials synthesis

The achiral diacrylate C6M (Wilshire Technologies), the right-handed chiral diacrylate monomer SLO4151 (Alpha Micron), and the right-handed chiral dopant R811 (EMD Chemicals) were used as received. Blue-phase LCE mixtures were prepared by adding 51 wt% C6M, 19 wt% SLO4151, 15 wt% R811, 14 wt% BDMT (benzenedimethanethiol, Sigma Aldrich), and 0.5 wt% Omnirad 819 (IGM Resins).

Alignment cells were made by spin coating two Corning EXG glass slides with 22 μL of photoalignment dye PAAD-22 (Beam Co.) diluted (2 to 1 by volume) with DMF. The coated glass slides were dried at 100 °C for 30 min to drive off any residual solvent. Once dried, the coated sides of two glass slides adhered together with an epoxy adhesive mixed with 20-μm glass spacers. Photoalignment was achieved by irradiating the alignment cell with a linearly polarized 445 nm laser at an intensity of 10 mW/cm^2^ for 10 min. Spatial variation in alignment utilized a photomask.

To prepare the liquid crystal elastomers retaining the various phases detailed hereto, the monomer mixture was melted to an isotropic phase at 90 °C and filled via capillary action into a 20-μm photopatterned alignment cell. The filled cell was then cooled to the desired temperature (phase) using an Instec HCS 402 heat stage. The filled cells were cooled at 2 °C/min to 70 °C, then 0.5 °C/min to 60 °C, and 0.25 °C/min at 0.5 °C increments thereafter. Samples were equilibrated at the designated temperature for at least 2 min. Thereafter, the mixtures were photopolymerized for 10 min with 365 nm light at an intensity of 50 mW/cm^2^. The polymer networks were extracted from the alignment cell and subsequently washed with acetone to remove any unreacted components.

### Materials characterization

Phase transition temperatures and micrograph textures were obtained via polarized optical microscopy (POM) on a Nikon Eclipse Ci-POL in reflection with inline Instec HCS 402 heat stage. Kossel diagrams were captured on the same microscope with 100× oil-immersion objective (NA = 1.25), Bertrand lens, and ${\lambda }_{c}$  = 440 × 10 nm bandpass filter.

Stress–strain measurements were performed on an RSA-G2 solids analyzer at a linear strain rate of 5% per minute. Glass transition temperatures were determined via differential scanning calorimetry (DSC) (TA Instruments Discovery DSC 2500). Transmission and reflection spectra of liquid crystal elastomers were collected with a Cary 7000 spectrophotometer (UV/Vis).

Gel fractions were obtained by recording the mass of the polymer sample before and after washing with dichloromethane for 24 h.

## Supplementary information

Supplementary information

Description of Additional Supplementary Files

Supplementary Video

## Data Availability

The data that support the findings of this study are available from the corresponding author upon reasonable request.
