# Author Correction: Retention and deformation of the blue phases in liquid crystalline elastomers

**DOI:** 10.1038/s41467-022-30790-x

**Published:** 2022-05-31

**Authors:** Kyle R. Schlafmann, Timothy J. White

**Affiliations:** 1 Department of Chemical and Biological Engineering, University of Colorado, Boulder, CO USA; 2 Department of Materials Science and Engineering, University of Colorado, Boulder, CO USA

**Keywords:** Liquid crystals, Liquid crystals

Correction to: *Nature Communications* 10.1038/s41467-021-25112-6, published online 13 August 2021.

The original version of this Article contained an error in the last two paragraphs in the ‘Results and Discussion’ section. The following sentences were incorrectly duplicated.

‘The branched, lightly cross-linked polymer network prepared from the liquid crystalline monomer mixture reported here can retain the three-dimensional, nanostructured architecture associated with either the body-centered cubic BPI or the simple cubic BPII. Mechanical, thermal, and chemical stimuli uniquely affect the lattice constants and the associated optical properties of these free-standing and fully solid optical materials. Whether the stimuli exposure introduces symmetric (heat, swelling) deformation or asymmetric (mechanical force) deformation of the lattice of the blue phases, in all cases the distortion is reversible.’

The correct version removes these sentences. The error has been corrected in both the PDF and HTML version of the Article.

