# Supplementary material for: Retention and deformation of the blue phases in liquid crystalline elastomers
Source: Nat Commun. 2021 Aug 13;12:4916. doi: 10.1038/s41467-021-25112-6 (PMC8363666; doi:10.1038/s41467-021-25112-6)
Supplement: Supplementary file 1 — Supplementary information [file 41467_2021_25112_MOESM1_ESM.pdf]

Retention and Deformation of the Blue Phases in Liquid Crystalline Elastomers

Kyle R. Schlafmann[1], Timothy J. White[1,2,*]

[1] Department of Chemical and Biological Engineering, University of Colorado – Boulder

[2] Materials Science and Engineering Program, University of Colorado – Boulder

* Correspondence to TJW - Timothy.J.White@colorado.edu

Supplementary Information

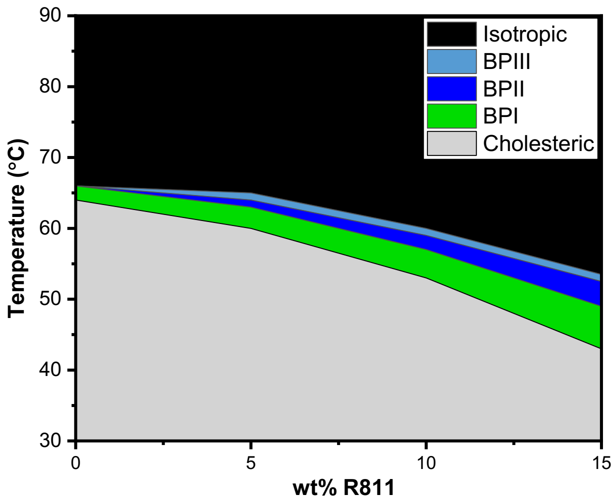

Supplementary Figure 1. **Blue phase stabilization.** Thermotropic phase windows for monomer mixtures (0.8:1 thiol to acrylate stoichiometric ratio) with constant weighted average helical twisting power. The chiral diacrylate SLO4151 has a helical twisting power of 8μm^{-1} and the non-reactive chiral dopant R811 has a helical twisting power of 11μm^{-1}).

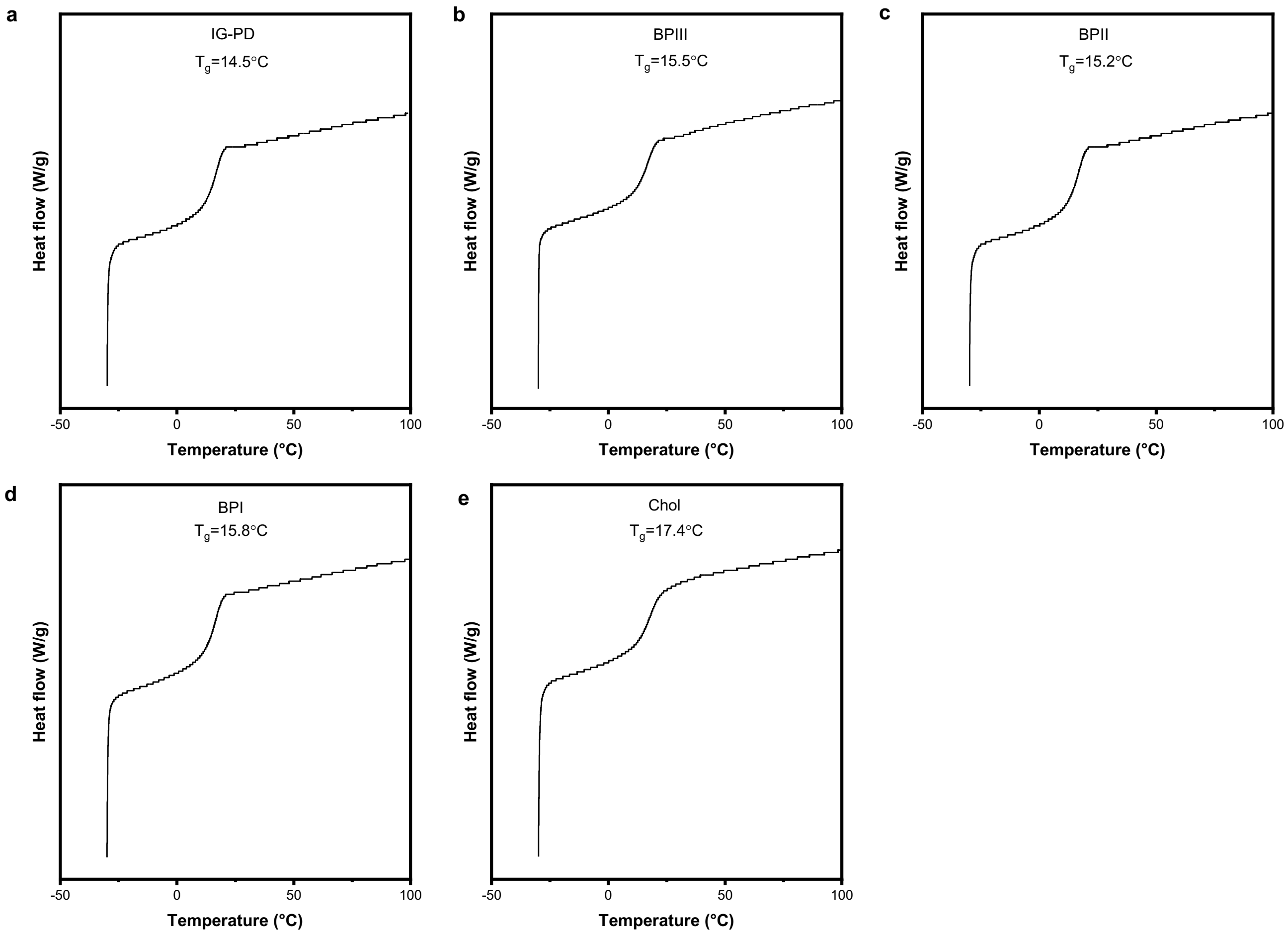

Supplementary Figure 2. **Glass transition temperatures of LCEs. a** Isotropic genesis polydomain nematic. **b** Blue phase III. **c** Blue phase II. **d** Blue phase I. **e** Cholesteric phase. Glass transition temperatures measured at the midpoint of the inflection point of the second heating curve (5°C/min) of a heat-cool-heat cycle.

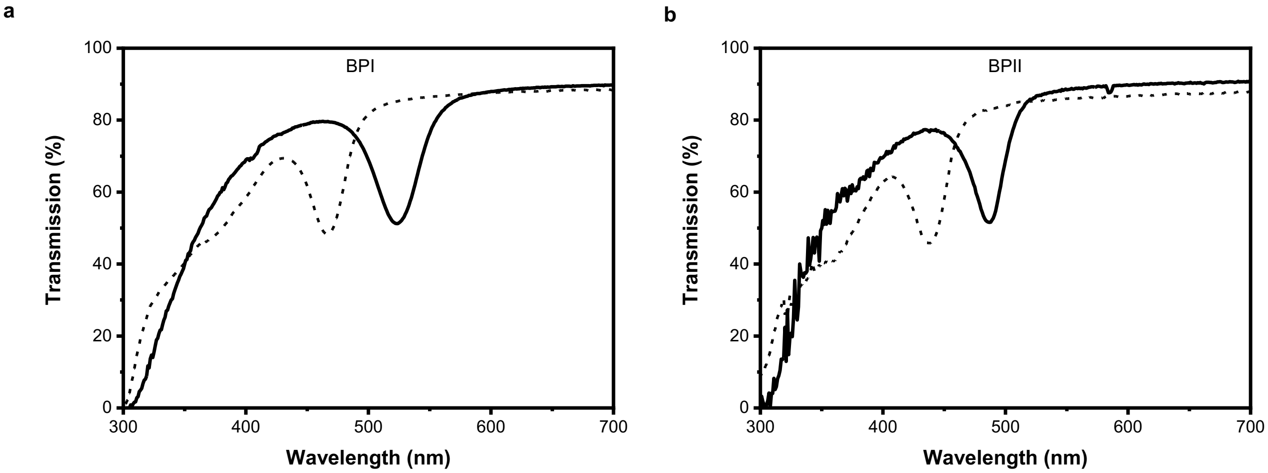

Supplementary Figure 3. **Wash effects. a** UV/Vis spectra of blue phase I elastomer (BPI LCE) before film extraction and solvent wash (solid line) and after (dashed line). **b** UV/Vis spectra of blue phase II elastomer (BPII LCE) before film extraction and solvent wash (solid line) and after (dashed line).

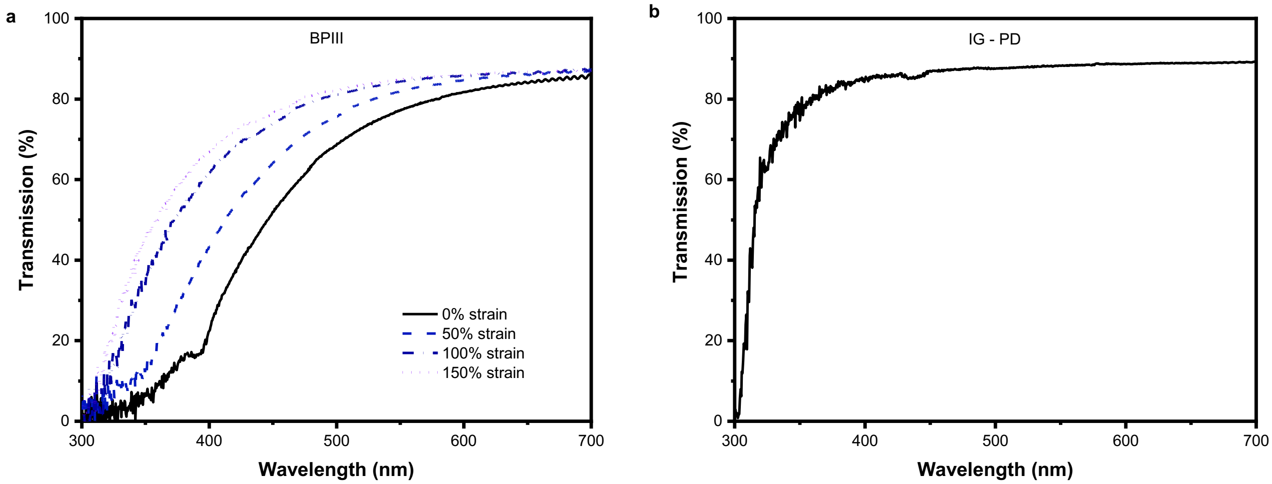

Supplementary Figure 4. **Spectra of amorphous phases. a** UV/Vis spectra of blue phase III elastomer (BPIII LCE) as a function of uniaxial strain. **b** UV/Vis spectra of isotropic genesis polydomain nematic elastomer (IG-PD LCE).

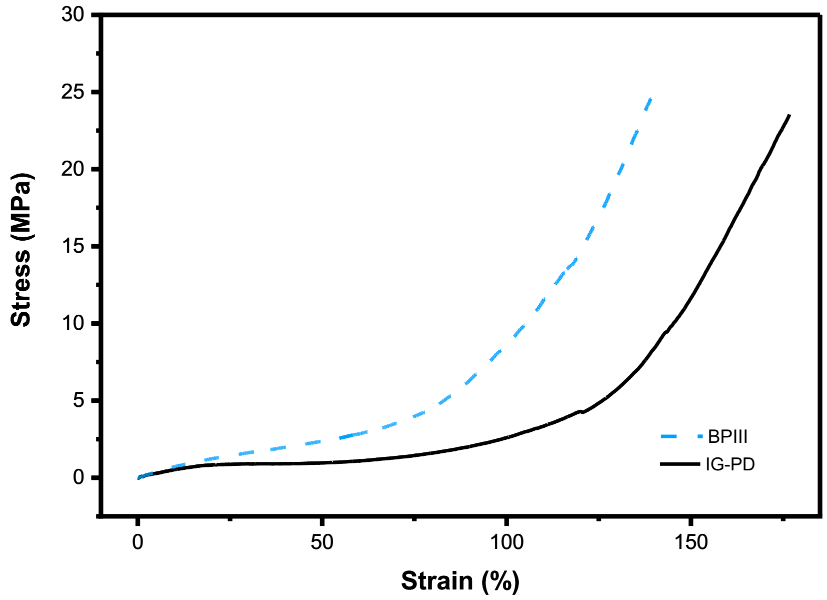

Supplementary Figure 5. **Mechanics of amorphous phase elastomers**. Stress-strain behavior of blue phase III elastomer (BPIII LCE) and isotropic genesis polydomain nematic elastomer (IG-PD LCE)

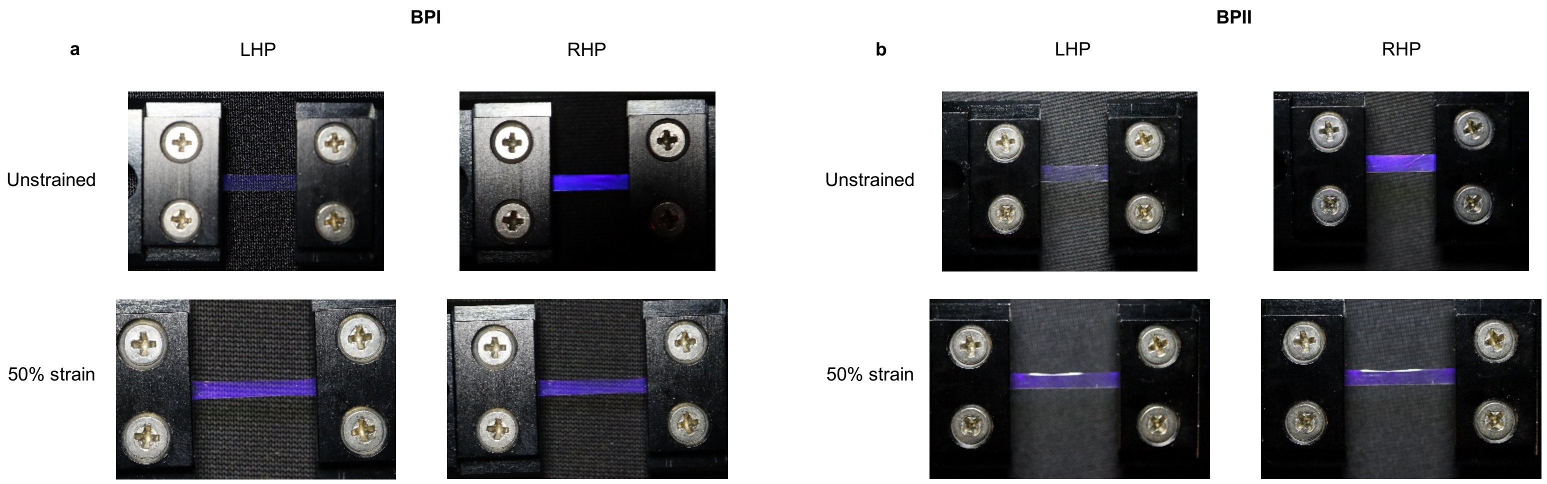

Supplementary Figure 6. **Reflection depolarization. a** Photographs of blue phase I elastomer (BPI LCE) illuminated by right-handed or left-handed circular polarization, with and without uniaxial deformation. **b** Photographs of blue phase II elastomer (BPII LCE) illuminated by right-handed or left-handed circular polarization, with and without uniaxial deformation.

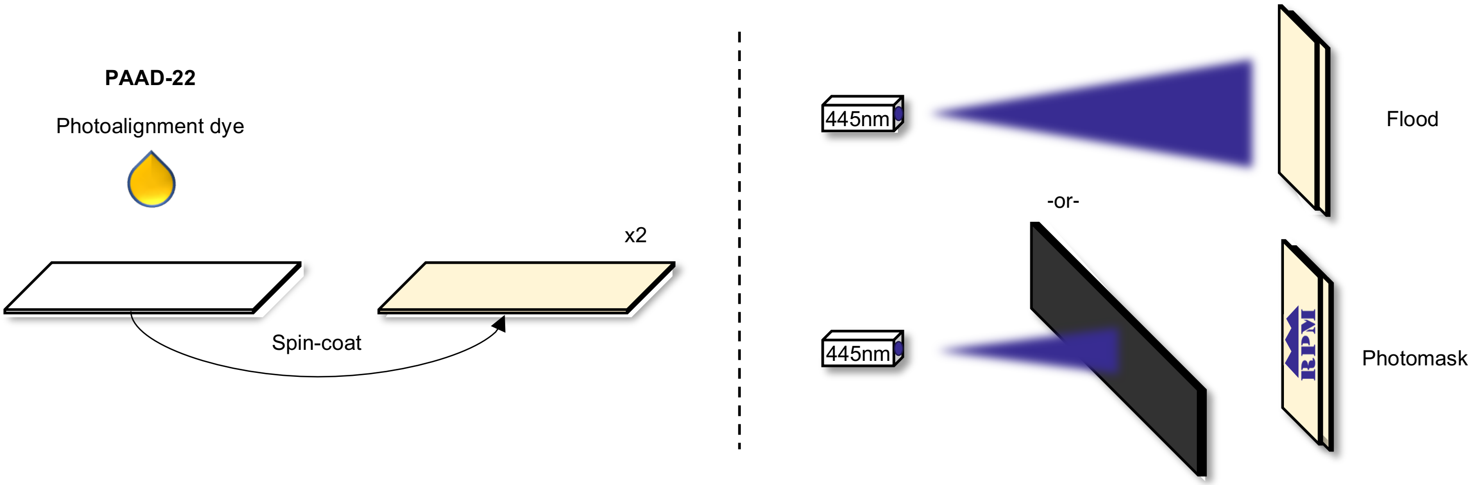

Supplementary Figure 7. **Photoalignment method.** Visual depiction of photoalignment cell preparation. Subsequent capillary fill of monomer mixture, cooling, and photopolymerization results in a photopatterned blue phase LCEs.
