# Supplementary material for: Retention and deformation of the blue phases in liquid crystalline elastomers
Source: Nat Commun. 2021 Aug 13;12:4916. doi: 10.1038/s41467-021-25112-6 (PMC8363666; doi:10.1038/s41467-021-25112-6)
Supplement: Supplementary file 2 — Description of Additional Supplementary Files [file 41467_2021_25112_MOESM2_ESM.pdf]

Description of Additional Supplementary Files

File name: Supplementary Movie 1 description
Description A Blue Phase II elastomer is exposed to dichloromethane under polarized optical microscopy. The polymer swells, resulting in a red-shift of the observed color from blue to infrared (appearing colorless). As the solvent evaporates, the polymer de-swells and the original color is restored.
